# The Statistical Report of King Edward's Hospital Fund
*Published by Spottiswoode and Co., Ltd., 5 New Street Square, E.C. Price 1s. net, 1s. 3d. post free.


**Published:** 1913-11-29

**Authors:** 


### HOSPITAL ECONOMICS.

### I.—The Statistical Report of King Edward's Hospital Fund.*

The ordinary man or woman, hearing that during last year the hospitals of London received well over five million attendances of out-patients, accepts the fact with a polite expression of interest and probably does not give the matter another thought. Even the hospital worker, counting over the cyphers in the stated total and finding them somewhere near his own estimate, is content to let the figures go without further examination.

Statistics seldom receive the attention the labour of compilation should win for them, and it is almost only when figures for comparison are set side by side that the calculations are examined with any thoroughness; even then the work their preparation has entailed is often not properly valued. There is a philanthropist who deserves to rank as high as the cheerful giver, and he is the careful giver. But no benefactor who makes use of organised charity can exercise care to a sufficient extent unless he is able to consult comparative figures. Should he refer to statistics of his own compilation, it is after infinite expenditure of time and trouble; therefore he must be glad to have at hand the requisite information on which to base his charity.

In respect of general charity, and particularly of hospitals, "Burdett's Hospitals and Charities" has for a quarter of a century proved the principal *vade mecum*, to which as a companion and not a competitor has been provided in recent years, for the use of the giver and the hospital worker, the specialised report of King Edward's Hospital Fund on the ordinary expenditure of the London hospitals.

This statistical report for the year 1912, dealing with the returns of 109 hospitals, makes the tenth annual contribution of the kind to the literature of hospital statistics issued from the office of King Edward's Fund. Its interesting history is well known to officers of hospitals. The first report was prepared by Mr. J. Danvers Power, then an hon. secretary of the Fund, in 1903; it referred to the figures of 16 hospitals. The earlier issues contained confidential matter, but, in showing the growth of the work, it is possible to give some indication of the general information afforded. For 1904 the issue referred to 36 hospitals, and for 1903 gave general particulars of numbers of patients and of the ordinary expenditure under the headings of the system of accounts then uniformly used. For 1905 and 1906 the report contained the same information extended to 48 hospitals, and for 1907 to 66 hospitals.

In 1908 the report was issued to the public for the first time. This publication followed on the revision of the Uniform System of Hospital Accounts. The old form had been a faithful servant for many years, but in view of several considerations it was thought desirable to make some alterations in order to arrive at a fairer means of comparison. Accordingly Mr. J. G. Griffiths, F.C.A., representing King Edward's Hospital Fund, sitting with a number of hospital secretaries and Messrs. R. A. Outhwaite and W. G. Burns, respectively representing the Hospital Sunday and Saturday Funds, were asked to undertake the work of revision, with the result that the forms at present used in presenting statements of income and expenditure, the balance-sheet, and other statistics, were drawn up.

The use of the system from January 1, 1907, has been necessary in the cases of all hospitals applying for grants from the three central funds. Some of the chief features of the forms are:—

(1) The statement of income and expenditure is no longer merely an analysis of the cash of the year, but of the income and expenditure, whether the cash is actually received and paid during the year or not.

(2) Methods of counting in-patients, out-patients, and out-patient attendances are improved.

(3) A more uniform plan of separating the cost of in- and out-patients leads to the production of a figure of increased reliability as to the cost per occupied bed in a given period.

(4) Building improvements are no longer included in the income and expenditure account; their cost is shown in the balance sheet.

(5) An improved method of stating the expenditure on laundry, either in or out of the hospital, is employed.

(6) The regular publication of a balance sheet and list of investments is required.

Such are the more important features of the revision; in presenting his report to King Edward's Fund, Mr. Griffiths stated that the system claimed no finality. This was as it should be; finality was not claimed for the forms of accounts previously used, and time brought about their revision. As the years pass by further revision will be required, especially in view of the changes in social conditions we now experience, and are, no doubt, destined to witness.

The use of the new forms brought material of a more reliable nature for the preparation of the Report for 1908, in which the compilers were content still to deal with sixty-six hospitals. For 1909 there was an extension to ninety-nine institutions, while from 1910 to 1912 the number dealt with was brought up to 109—practically all the hospitals receiving grants from King Edward's Fund.

The chief possibility given by the revision was fearless comparison of the cost per bed and the cost per out-patient attendance. This process has been freely made use of in the Report since 1908, and the tables given in the last issue are as follows:—

(1) Introductory tables concerning the whole of the 109 hospitals under review, giving (*a*) general particulars; (*b*) total ordinary expenditure; (*c*) cost per bed and cost per out-patient attendance.

(2) Group comparisons under six headings of expenditure: (*a*) Larger General Hospitals;

* Published by Spottiswoode and Co., Ltd., 5 New Street Square, E.C. Price 1s. net, 1s. 3d. post free.

(*b*) Smaller General Hospitals; (*c*) Consumption Hospitals; (*d*) Hospitals for Women; (*e*) Children's Hospitals; (*f*) Ophthalmic Hospitals; (*g*) Hospitals for Nervous Diseases; (*h*) Cottage Hospitals; (*i*) Lying-in Hospitals.

(3) Analysis of cost of washing at 106 hospitals.

(4) Table showing the highest and lowest prices paid for supplies of various goods on Dec. 31, 1912.

In the group comparisons we are given not only the cost per occupied bed and out-patient attendance, but are shown with much ingenuity the total value of the divergence in each case from the average, instances of which we shall examine in a later article.

Before we touch upon other matters, it is a pleasure and a duty to say a word of recognition of the labour of those whose efforts have provided the hospital world with an instrument of such utility. Mr. Danvers Power was identified with the inception; Mr. H. R. Maynard, the Secretary of King Edward's Fund, has carried on and extended the Report in recent years; and by the secretaries and accountants of the various hospitals the material for the Report is cheerfully given at the cost of some labour and time.

It should be stated that previous to 1907 there was no suggestion that an unfair advantage was sought or gained in any case because of the greater licence of the older system. The immediate result of the change as shown by the figures of 1906 and 1907 indicates that, generally speaking, there was a fall all round in the cost per occupied bed under the headings used, now lightened by the elimination of items the cost of which is not under control from year to year. There are, it is true, those cases of strange variation from a general rule which are a feature of hospital finance.

*(To be continued.)*

